# Environmental changes to reduce self-harm on an adolescent inpatient psychiatric ward: an interrupted time series analysis

**DOI:** 10.1007/s00787-020-01607-3

**Published:** 2020-07-27

**Authors:** Gurpreet K Reen, Jill Bailey, Lorna McGuigan, Natasha Bloodworth, Rasanat Fatima Nawaz, Charles Vincent

**Affiliations:** 1 Department of Experimental Psychology, University of Oxford, Oxford, OX2 6GG UK; 2 Oxford Healthcare Improvement Centre, Oxford Health NHS Foundation Trust, Oxford, OX3 7JX UK; 3 Patient Safety Collaborative, Oxford Academic Health Science Network, Oxford, OX4 4GA UK

**Keywords:** Self-harm, Inpatient, Mental health, Adolescent, Children, Psychiatry

## Abstract

Existing interventions to reduce self-harm in adolescents admitted to psychiatric wards are usually focused on individual psychological treatments. However, the immediate ward environment in which treatment takes place is an important factor in the success of the treatment and can also influence the likelihood of self-harming behaviours. The aim of the current study was to evaluate changes made to a psychiatric ward environment on incidence of self-harm in adolescents. A quasi-experimental interrupted time series study was conducted on one child and adolescent psychiatric ward. An intervention was developed alongside staff and patients to address the high incidence of self-harm on weekday evenings on the ward. The intervention components involved adding a regular twilight shift (3–11 pm) for nursing staff and introducing a structured evening activity programme on the ward. A segmented regression analysis of an interrupted time series found that the rate of self-harm per 100 bed days was already declining at baseline and continued to decline post-intervention, but the rate of decline was not significant (*p* = 0.415). However, the proportion of patients self-harming was increasing at baseline and significantly reduced post-intervention (*p* = 0.001), and this reduction was significantly larger in the evenings (*p* = 0.004) compared to other times of day (*p* = 0.09). A tailored intervention targeting the psychiatric ward environment helped to reduce the proportion of adolescents self-harming on the ward. An interrupted time series analysis should be considered for future interventions making changes to health systems over time.

**Electronic supplementary material:**

The online version of this article (10.1007/s00787-020-01607-3) contains supplementary material, which is available to authorized users.

## Introduction

Self-harm, also referred to as deliberate self-harm, describes the action of intentionally injuring or poisoning oneself regardless of motivation or suicidal intent [1, 2]. Functions that may motivate or reinforce non-suicidal self-harming behaviour are self-punishment and avoidance of negative emotions [3–6]. Other functions of self-harm have also been identified such as interpersonal influence (to seek help from others) and peer bonding (fitting in with others), as well as sensation-seeking (seeking excitement, anti-dissociation) and gratification (self-harming as comforting) [3, 4]. Functions of self-harm with suicidal intent also include many functions associated with non-suicidal self-harm behaviour, in particular coping with self-hatred and sensation seeking [7]. These functions are exhibited in adolescents, and when combined with factors such as impulsivity and exposure to others who self-harm, can translate into self-harming behaviour with or without suicidal intent in the younger population [1, 8].

Adolescents with complex mental health needs and who are at serious risk of harming themselves or others may be supported in the community in services such as intensive home treatments and specialist outpatient settings [9–11]. However, some of these patients will be admitted for care in inpatient psychiatric wards [11, 12]. Around 10–20% of adolescents on inpatient wards will self-harm at least once during their stay, and a proportion of these will self-harm repeatedly as many as 130 times [13–16]. Longer duration on an inpatient ward is also predictive of more self-harm incidents in adolescents, and therefore, it is essential that adolescents should be supported in this setting [14]. Self-harm can have a damaging physical and psychological impact on the young person harming and also negatively impacts others who encounter the incident on a psychiatric ward. Clinicians on adolescent psychiatric wards report feeling distressed when attempting to de-escalate a self-harm attempt [17], in particular if using a restrictive practice [18–20]. Other adolescents on the ward are often distressed and influenced by self-harming behaviour and must find ways to cope with these feelings [21, 22]. Reducing self-harm on adolescent psychiatric wards is necessary to improve the well-being of the young person who self-harms, as well as other patients and clinicians present in this secure environment.

### Risk factors of self-harm on inpatient psychiatric wards

Many adolescents on inpatient psychiatric wards have a previous history of self-harm. The risk factors of self-harm with both non-suicidal and suicidal intent are complex and include age, gender, mental health diagnosis, coping strategies, previous self-harm, acute stress response, relationship with family and friends, as well as social deprivation [7, 23–27]. The likelihood of self-harm is also influenced by more immediate contextual factors such as social influences and the environment of care on inpatient psychiatric wards [12, 23, 28, 29].

Young people may be inclined to self-harm by mimicking self-harming behaviours in others [1]. This is of particular concern on inpatient psychiatric units, where young people are in close proximity to others who self-harm [15, 30, 31]. However, such contagion effects are only one possible environmental influence. A combination of loneliness, isolation from others and a lack of stimulation can further contribute to self-harming behaviours of inpatients on psychiatric wards [22, 32]. This is likely due to an increase in negative emotions in an individual, as well as the positive functions of self-harm which can provide comfort and excitement during this period [4]. This is apparent on adult psychiatric wards, where self-harming behaviours commonly occur when patients are alone in the evening [32–34] and on private areas like the bedroom and bathroom [32, 33]. Young people in the community also report more self-harming thoughts when they are alone [35] and usually consider self-harm a private act, to be done in secrecy [36]. The social relationship between adolescents and nursing staff on psychiatric wards can also influence self-harming behaviours, as young people reportedly harm less when nursing staff intervened during early warning signs of distress [28]. As indicated by the interpersonal function of self-harm, it is possible that patients may use self-harming behaviour to seek help when they do not feel supported by nursing staff on wards [3, 5]. Other contextual factors such as interaction with other inpatients, ward rules and routines, length of stay on the ward, amount of leave granted, voluntary or involuntary admission, and the general ward atmosphere are also likely to contribute to self-harming behaviours in adolescents admitted on inpatient psychiatric wards [12, 23, 28].

### Interventions to reduce adolescent self-harm on wards

Therapeutic approaches such as dialectical behaviour therapy (DBT) and cognitive behaviour therapy (CBT) are commonly used to treat adolescents who self-harm with and without suicidal intent. DBT is a highly structured psychotherapy delivered in individual sessions and in groups to help patients regulate their emotions and equip them with the skills to tolerate distress, practice mindfulness and reduce maladaptive behaviours such as self-harm [37, 38]. CBT similarly helps patients to regulate their emotions by modifying distorted thinking patterns and strengthening coping, communication and problem solving skills [39, 40]. DBT and CBT have been adapted and implemented within inpatient settings and have reduced self-harming behaviours in adolescents [37, 39–43]. However, the ward environment also plays a key role in the success of these treatments and can actively contribute to self-harming behaviours in adolescents [12, 23, 28, 29]. Safety analysis in other areas of healthcare, and in other industries, also suggest that wider organisational and environmental factors are important contributory factors to safety incidents [44]. Thus, interventions to reduce self-harm in adolescents should not just treat the young person but consider improving the immediate psychiatric ward environment for adolescents being cared for in this setting.

A small number of interventions have combined therapeutic interventions with environmental changes on the ward to reduce self-harming behaviour. In one study, adolescent inpatients were exposed to either normal DBT training, DBT-based environmental changes or both [45]. The environmental changes included analysing problematic behaviour in patients and behavioural interventions [45]. However, only non-suicidal self-harm incidents were monitored in this study and the effects on self-harm were unclear due to high levels of attrition [45]. More recently, adolescent inpatients were given extensive DBT skills training as well as being introduced to daily leisure activities on the ward such as pet therapy and pottery making [46]. The intervention was successful in reducing both suicidal and non-suicidal self-harm when compared to adolescents treated as usual, but the influence of activities as an environmental change on self-harm was unclear [46]. Meaningful activities have often been suggested as a possible way to distract inpatients who may self-harm on an inpatient setting [33, 36, 47] as distraction can help adolescents cope with distress when alone [6, 34, 48]. It is also possible that meaningful activities can serve to replace the positive functions associated with both non-suicidal and suicidal self-harm, such as feelings of gratification and sensation seeking [4]. This needs to be examined further on inpatient settings.

Other interventions have made changes to only the psychiatric ward environment to reduce self-harm in patients on the ward. One study found that employing an additional nurse to improve communication between staff and inpatients and ensure an ethical approach to rules and routines helped reduce harmful patient behaviours including self-harm [49]. Another intervention, consisting of ten best practices for staff to communicate with inpatients, also showed a significant reduction in self-harming behaviours [50]. However, these and other similar interventions have been conducted on adult inpatient settings [49–51] and have not always been replicated [52]. A much larger intervention conducted over 5 years on an adolescent psychiatric unit significantly reduced self-harm by training staff to improve communication with adolescents on the ward and by improving responses to maladaptive patient behaviours [53]. Staff training and better responses to harmful patient incidents was also found to be effective in reducing aggressive incidents in adolescents on a psychiatric ward, including aggression towards themselves [54]. While these interventions are very important in the longer term care of patients, they do not offer immediate improvements to the ward environment to reduce self-harm in adolescents.

Environmental changes that do successfully reduce self-harm for both adults and adolescents admitted on a psychiatric ward have generally been analysed using a simple pre–post analysis [49, 50, 54]. This analysis does not take into account the longitudinal nature of these changes nor does it consider any pre-intervention trends; for instance, if harmful incidents were reducing before the intervention then a pre–post analysis could show a significant decrease in incidents even when this does not actually exist (i.e., a type 1 error). An interrupted time series analysis is an alternative approach which takes into account both the longitudinal data and pre-intervention trends and should be considered when evaluating health system interventions over time [55, 56].

### Objective

The current intervention was designed to improve an adolescent psychiatric ward environment with input from staff and patients on the ward. The aim of the intervention was to (i) reduce the rate of self-harm incidents and (ii) reduce the proportion of adolescents self-harming on the ward, by rigorously evaluating the intervention using an interrupted time series analysis.

## Methods

### Study design

A quasi-experimental design using an interrupted time series analysis was conducted to evaluate an intervention that made changes to the psychiatric ward environment. The baseline period was 1st June 2016 to 31st May 2018 and the intervention was introduced on 1st June 2018. Outcome data post-intervention was collected for 18 months. The study was primarily aimed at improving a healthcare service, and therefore, a formal research ethics application was not required.

### Setting and participants

The study was carried out on one child and adolescent psychiatry inpatient ward in the UK for children aged between 12 and 18 years. The ward has 12 inpatient beds and has a school for patients on the ward to attend in the day. Visiting hours on the ward are usually between 4.30 and 8.30 pm in the evenings and many inpatients are also given leave from Friday evening to Sunday evening to be at home with their families.

Group therapy sessions happen daily between 2 and 3 pm, with some occasional activities in the evenings. Individual treatment sessions usually consist of weekly meetings with an assigned key nurse, psychiatrist and psychology sessions as needed. Patients with emotional dysregulation also attend the ‘managing emotions’ pathway, consisting of individual skill learning and weekly group sessions. Medication is provided to patients based on clinical need and within dose recommendations by the British National Formulary. This includes antidepressants as clinically required for depression, anxiety, panic or PTSD, low dose antipsychotic sometimes prescribed for agitation, and a low dose benzodiazepine during de-escalation, only if not possible to de-escalate with good nursing care, distraction or reinforcing of coping skills. Rapid tranquisalisation is rarely used on the ward.

The ward has a multidisciplinary team of staff who support the care of inpatients, including: 1 full time equivalent consultant psychiatrist, 2 trainee doctors, 0.6 speciality doctor, 0.8 family therapist, 0.5 social worker, 1 clinical psychologist, 1 assistant psychologist and 1.2 occupational therapists. Prior to the intervention, the regular shift patterns for nursing staff on the ward were early (7 am–2.45 pm), late (1.15 pm–9 pm) and night (8.40 pm–7.20 am), with 7 nurses on the ward during early/late and 5 nurses at night. An ad-hoc twilight shift (3–11 pm) was introduced on some evenings at short notice when the ward was considered unstable, and these would often be covered by expensive temporary nursing staff.

### Intervention

The intervention was co-designed with clinical ward staff with regular input from patients to reduce self-harm on the ward. The experiences of clinical staff and routinely collected self-harm data on the ward highlighted a clear temporal tend; 62% of self-harm incidents occurred between 5 pm and 11 pm over a year. With this insight, an intervention was designed to focus on the vulnerable evening period on the ward. Iterative changes were made to the intervention following feedback from staff and patients, but the main intervention components did not change.

#### Regular twilight shifts

The first component of the intervention was introducing a regular twilight shift for nursing staff (3 pm–11 pm, Sunday–Thursday) to provide additional support on the ward during the vulnerable evening period and during the transition of late shift to night shift staff. The regular twilight shifts were introduced from 1st June 2018. Although self-harm incidents on the ward were highest between 5 and 11 pm, the twilight shifts were kept at 8 h to comply with NHS guidelines. No twilight shifts were added on Fridays and Saturdays as many inpatients take leave from Friday evenings to Sunday afternoon. The intervention component was designed to increase availability of regular nursing staff on the ward during a vulnerable time, rather than employing expensive temporary agency staff. Although cost and travel implications made it challenging for regular nursing staff to take these shifts when first introduced, over time there was a gradual decrease of temporary staff being used on the ward as twilight shifts began to be filled by regular nursing staff (see Appendix Table A.1).

#### Evening activities

The second component of the intervention was a structured programme of evening activities. The evening activities were introduced gradually on the ward from 1st July 2018 with a complete programme available from 1st September 2018. These activities were not intended to be directly therapeutic, but simply normal activities for young people to take part in during less structured times of the day. All activities were voluntary. The attendance for each evening activity was not recorded. However, staff on this small inpatient unit made every effort to invite all patients to attend evening activities, and attendance was high at most activities. All patients on the ward attended evening activities during the course of their stay if they were well enough to join. Patients were encouraged to suggest activities they would like, and activities offered in the evening changed regularly to reflect their feedback. Activities included a games and drama workshop (e.g., role-playing and storytelling), visits from a Pets As Therapy (PAT) dog, mindfulness podcast groups, and an art and coping skills workshop (e.g., drawing, painting and pottery), conducted by activity workers or occupational therapists on the ward (see Table 1).

**Table 1:** Example of a structured evening activity programme on the ward

Evenings	Activity offered
Monday	Mindfulness podcast
Tuesday	Art and coping skills; mindfulness podcast*
Wednesday	PAT dog visit; mindfulness podcast*
Thursday	Games and drama workshop; mindfulness podcast*

All activities were an hour long and took place before and after evening dinner (between 5 and 9 pm)

*PAT* pets as therapy

*Patients could choose to attend either of the two activities offered

### Measures

Outcome measures were collected through routinely available data in the healthcare organisation. All data used in this study is routinely reported by clinical staff on the inpatient psychiatric ward through an incident reporting system. The data reported will include detailed information about the incident, such as the type of self-harm, the patient and staff involved, the harm to the patient or others, measures used to contain the self-harm and a narrative summary of the incident. The incident report is subsequently checked by the matron of the inpatient psychiatric ward, the system administrator of the incident reporting database, and the clinical lead for the organisation who is responsible for producing quarterly reports on self-harm as well as other major incidents. Although the clinical staff and the matron were not blinded to the intervention, other parties responsible for checking the data were not aware of when the intervention was happening on the ward. There was no change to routine data-reporting pre- and post-intervention.

The primary outcome measures were rate of self-harm per 100 bed days and the proportion of patients self-harming. Self-harm was defined as intentional self-poisoning or injury, irrespective of whether the act was intended as suicidal or non-suicidal. All types of self-harm were included, such as poisoning, asphyxiation, cutting, burning and other self-inflicted injuries. This definition was in line with the national guidance on how self-harm incidents should be recorded by healthcare organisations.

#### Rate of self-harm incidents per 100 bed days

Monthly number of self-harm incidents on the ward were collated between 1st June 2016 and 31st November 2019. A standardised self-harm rate per 100 occupied bed days was calculated (i.e., the number of self-harm incidents that occurred for every 100 days an inpatient was on the ward). This is a recommended method to report incidents as it takes into consideration the varying lengths of stay by patients and can also be easily compared to incidents on other wards [57]. The psychiatric ward in this study had an average bed occupancy rate of 75.5% between November 2017 and November 2019 (bed occupancy rate prior to these dates was not easily available). To calculate the rate of self-harm in June 2016 as an example, the number of incidents that occurred during this month was divided by the number of beds available that month ((12 beds × 30 days) × 75.5%), and then multiplied by 100. Monthly rates of self-harm per 100 occupied bed days was calculated overall and was also spilt by time of day to determine whether the reduction of self-harm was larger in the evening compared to other times of the day. For the purpose of this study, evening referred to 3–11 pm (to align with the twilight shift hours) and non-evening was any time of day excluding 3–11 pm.

#### Proportion of patients self-harming

The overall rate of self-harm is important but may be unduly influenced by a small number of people who self-harm very frequently [57]. From a therapeutic standpoint it is arguably even more important to reduce the number of people who self-harm. The number of patients self-harming on the ward each month were collated between 1st June 2016 and 31st November 2019. This was divided by the total number of patients that were admitted on the ward that month, and then multiplied by 100 to obtain the percentage of patients self-harming. This is a standardised measure that takes into account the different number of patients present on inpatient wards and can be compared across inpatient services [33, 57]. As well as the total proportion, the proportion of patients self-harming in the evening and non-evening period was also calculated.

#### Patient characteristics and diagnosis

Patient characteristics and clinical diagnosis were obtained from the hospital episodes statistics database from the healthcare organisation. Patient diagnosis was based on a full clinical assessment conducted by a consultant psychiatrist. These clinical assessments are based on the ICD-10 criteria [58], the clinical judgment of the psychiatrist and discussions with the patient and their family. When clinically indicated, the diagnosis for mental health patients is clearly described to patients and families, including for patients with emotionally unstable personality disorder. However, in cases when the symptomatology remains unclear, the diagnosis will be tentative and subject to review.

### Statistical analysis

Patient characteristics at baseline and post-intervention were analysed for differences using an ANOVA and Chi square analysis. A segmented regression analysis of an interrupted time series was conducted to compare monthly data on rate of self-harm and proportion of patients self-harming before and after the intervention was introduced, as recommended by previous studies [55, 56, 59–61]. The analysis was done for a 2-year baseline period (1st June 2016 to 31st May 2018) and 18-month post-intervention (1st June 2018 to 31st November 2019). It was expected that the intervention would have a gradual impact on the outcome of self-harm, and therefore, only the change in slope was analysed at baseline compared to post-intervention over time [55, 60].

All data was analysed using R software [62]. A Poisson regression model was used to analyse the rate of self-harm per 100 bed days by including the count of all self-harm incidents as a dependent variable in the model and the occupied bed days as an offset term. A Binomial regression model was used to analyse the proportion of patients self-harming. Autocorrelation in the data was assessed by examining the partial autocorrelation function and by conducting the Breusch–Godfrey test [63]. Autocorrelation refers to any significant correlation between data reported at one time point with subsequent time points (i.e., 1 month with any subsequent months). A significant correlation between every 12 months would indicate seasonality in the dataset. Minimal autocorrelation was identified for findings that were significant pre and post-intervention. Therefore, no adjustments for autocorrelation to these models were required. The counterfactual scenario, or the assumption that the pre-intervention trend would have continued unchanged if there was no intervention, was also computed. Two patients that self-harmed extensively (> 3.5 standard deviations over the mean self-harm incidents per person) were considered outliers in the study. Segmented regression analysis was conducted without the outliers and with the outliers included.

## Results

### Participants

A total of 205 young people were hospitalised for psychiatric care on one UK adolescent psychiatric ward between 1st June 2016 and 31st November 2019. Patients ranged from 12 to 18 years, and mean age was 15.65 years (SD 1.48). Average length of stay was 75.27 days (SD 72.27) and ranged from 0 to 406 days. The majority of patients were female (*n* = 175, 85.37%) and the remaining patients were male (*n* = 29, 14.15%) or did not specify their gender (*n* = 1, 0.49%). The most common primary mental health diagnosis was eating disorders (*n* = 87, 42.44%). Only 6 patients had an unspecified mental health disorder (2.92%).

There were 124 patients on the psychiatric ward before the intervention was implemented (1st June 2016 to 31st May 2018) and 71 patients after implementation (1st June 2018 to 31st November 2019). A further 10 patients remained on the psychiatric ward both before and after the intervention was introduced (see Table 2). There was no significant difference in age (*F* = 2.29, *p* > 0.05) and gender (*x*^2^ = 7.84, *p* > 0.05) between patients in either groups.

**Table 2:** Patient characteristics pre- and post-intervention

	Pre-intervention (*n* = 124)	Post-intervention (*n* = 71)	Pre- and post-intervention (*n* = 10)
Age, years			
Mean ± SD	15.81 ± 1.41	15.35 ± 1.60	15.90 ± 1.29
Range	12–18	12–18	14–18
Gender (*n*, %)			
Male	17 (13.7%)	8 (11.3%)	4 (40%)
Female	107 (86.3%)	62 (87.3%)	6 (60%)
Not specified	0	1 (1.4%)	0
Length of stay, days			
Mean ± SD	64.29 ± 65.07	81.97 ± 67.51	163.80 ± 119.77
Range	0–328	5–298	62–406
Primary diagnosis (*n*, %)			
Adjustment and dissociative	6 (4.8%)	2 (2.8%)	0
Anxiety	11 (8.9%)	7 (9.9%)	0
Developmental	3 (2.4%)	2 (2.8%)	1 (10%)
Eating	46 (37.1%)	35 (49.3%)	6 (60%)
Mood	19 (15.3%)	9 (12.7%)	0
Obsessive compulsive	1 (0.8%)	1 (1.4%)	1 (10%)
Other	9 (7.3%)	5 (7.0%)	1 (10%)
Personality	8 (6.5%)	4 (5.6%)	0
Phobias	1 (0.8%)	0	1 (10%)
Schizophrenia and psychosis	9 (7.3%)	2 (2.8%)	0
Stress-related	2 (1.6%)	1 (1.4%)	0
Substance abuse	3 (2.4%)	1 (1.4%)	0
Unknown	5 (4.0%)	1 (1.4%)	0

Pre-intervention dates: 1st June 2016 to 31st May 2018; Post-intervention dates: 1st June 2018 to 31st November 2019; Pre- and post-intervention dates: 1st June 2016 to 31st November 2019

### Impact of intervention on rate of self-harm

The average rate of self-harm per 100 bed days per month shows that self-harm incidents reduced post-intervention compared to baseline (see Table 3). When split by time of day, the average rate of self-harm per month also showed a reduction both in the evening and non-evening period following the intervention compared to baseline.

**Table 3:** Rate of self-harm per 100 bed days per month pre- and post-intervention, without outliers

	Self-harm incidents, total		Self-harm incidents, evening		Self-harm incidents, non-evening	
	Mean (SD)	Range	Mean (SD)	Range	Mean (SD)	Range
Pre-intervention	5.49 (3.47)	1.07–13.61	3.58 (2.36)	1.07–9.20	1.91 (1.34)	0–4.42
Post-intervention	3.23 (2.27)	0–9.20	2.21 (1.81)	0–7	1.02 (0.93)	0–2.94

Pre-intervention dates: 1st June 2016 to 31st May 2018; Post-intervention dates: 1st June 2018 to 31st November 2019; Evening = 3–11 pm; Non-evening = any time excluding 3–11 pm

**Fig. 1:**
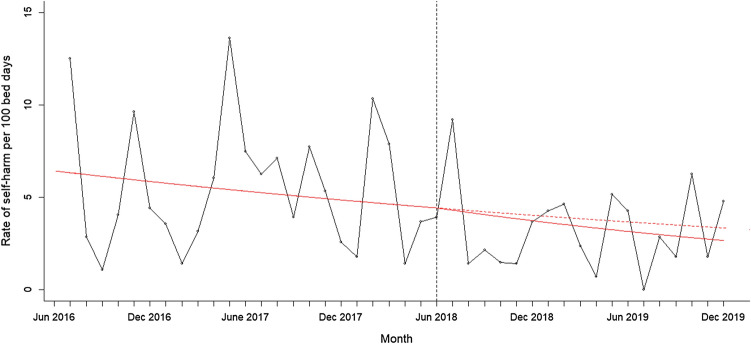
Rate of self-harm per 100 bed days at baseline and post intervention. The figure shows the monthly rate of self-harm between 1st June 2016 and 31st November 2019 after removing outliers. The intervention was introduced on 1st June 2018 indicated by the vertical line. The solid red line indicates the segmented regression analysis conducted at baseline and at post-intervention. The red dashed line indicates the counterfactual scenario (i.e. projected rate of self-harm if the intervention had not been conducted)

**Fig. 2:**
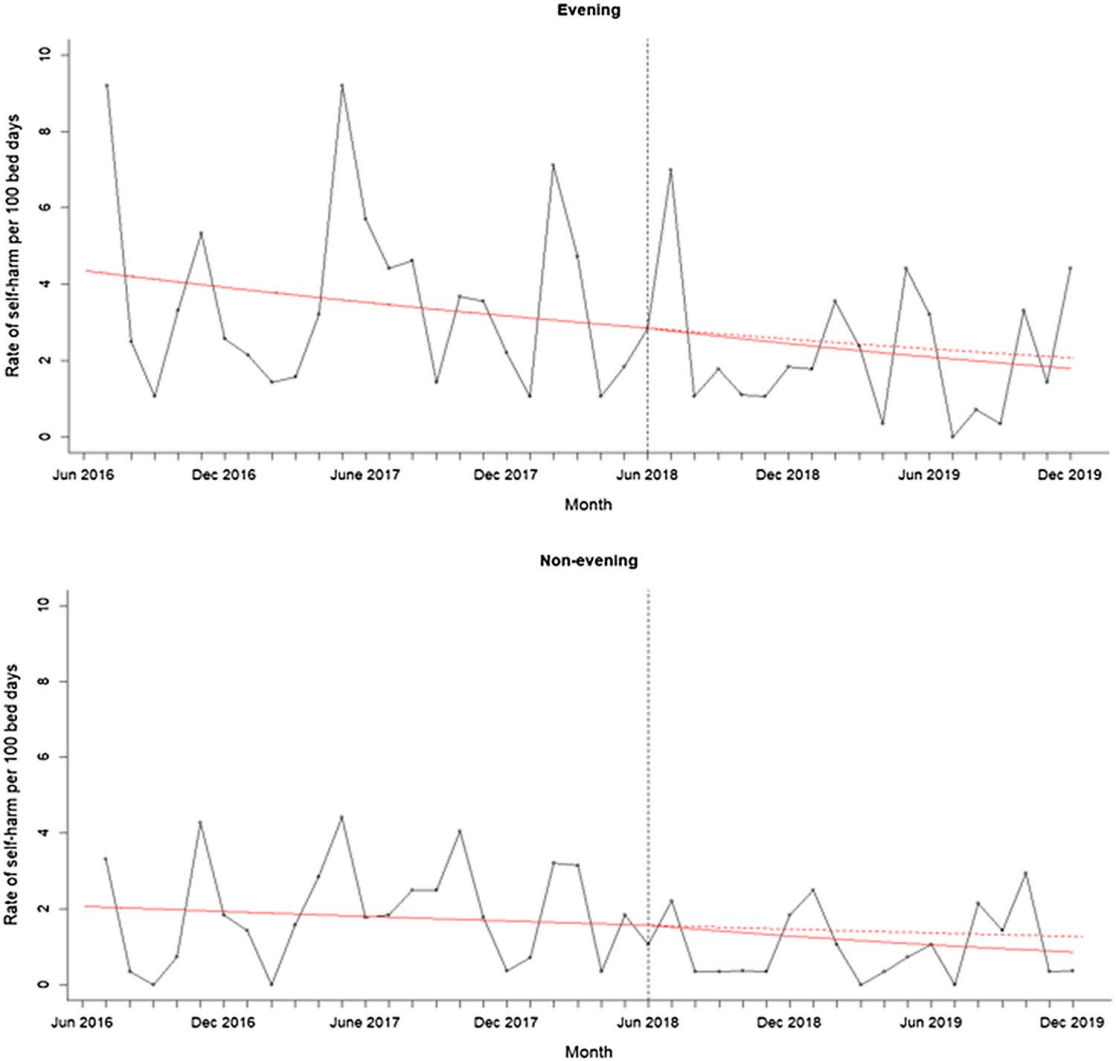
Rate of self-harm per 100 bed days at baseline and post intervention split by evening (3-11pm) and non-evening period. The figure shows the monthly rate of self-harm between 1st June 2016 and 31st November 2019 after removing outliers, split by time of day. Evening refers to 3-11pm and non-evening period refers to any time of day excluding 3-11pm. The intervention was introduced on 1st June 2018 indicated by the vertical line. The solid red line indicates the segmented regression analysis conducted at baseline and at post-intervention. The red dashed line indicates the counterfactual scenario (i.e. projected rate of self-harm if the intervention had not been conducted)

A segmented regression analysis for monthly rates of self-harm per 100 bed days without outliers showed that the rate of self-harm was steadily declining before the intervention was implemented, but the rate of decline was not significantly affected by the intervention (see Fig. 1; change in slope – 0.01, 95% CI – 0.04 to 0.02, *p* = 0.415). When split by time of day, the rate of self-harm was declining in the evening and non-evening period before the intervention and again the decline was not significantly affected by the intervention (see Fig. 2; Evening: change in slope – 0.007, 95% CI – 0.05 to 0.03, *p* = 0.676; Non-evening: change in slope – 0.02, 95% CI – 0.07 to 0.03, *p* = 0.414). This analysis shows that while the rate of self-harm continued to decline on the psychiatric ward after the intervention was introduced, this was not significantly affected by the evening-based interventions.

When outliers were included in the analysis, the monthly rates of self-harm per 100 bed days was also steadily declining before the intervention but the rate of self-harm significantly increased following the intervention (change in slope: 0.09, 95% CI 0.07–0.11, *p* < 0.0001). When split by time of day, the rate of self-harm was declining in the evening and non-evening period before the intervention and again the rate significantly increased following the intervention (Evening: change in slope 0.09, 95% CI 0.06–0.12, *p* < 0.001; Non-evening: change in slope 0.08, 95% CI 0.04 to 0.12, *p* < 0.001). This indicates that the rates of self-harm increased post-intervention mostly due to two patients who self-harmed frequently.

### Impact of intervention on proportion of patients self-harming

The average proportion of patients self-harming per month reduced post-intervention compared to baseline (see Table 4). When split by time of day, the average proportion of patients self-harming also reduced both in the evening and non-evening period following the intervention compared to baseline.

**Table 4:** Proportion of patients self-harming per month pre- and post-intervention, without outliers

	Patients self-harming, total		Patients self-harming, evening		Patients self-harming, non-evening	
	Mean (SD)	Range	Mean (SD)	Range	Mean (SD)	Range
Pre-intervention	33.09 (13.94)	12.50–73.33	26.50 (11.46)	6.67–60.00	17.81 (11.59)	0–46.67
Post-intervention	20.35 (20.35)	0–40.00	17.19 (10.11)	0–33.33	8.69 (6.27)	0–26.67

Pre-intervention dates: 1st June 2016 to 31st May 2018; Post-intervention dates: 1st June 2018 to 31st November 2019; Evening = 3–11 pm; Non-evening = any time excluding 3–11 pm

**Fig. 3:**
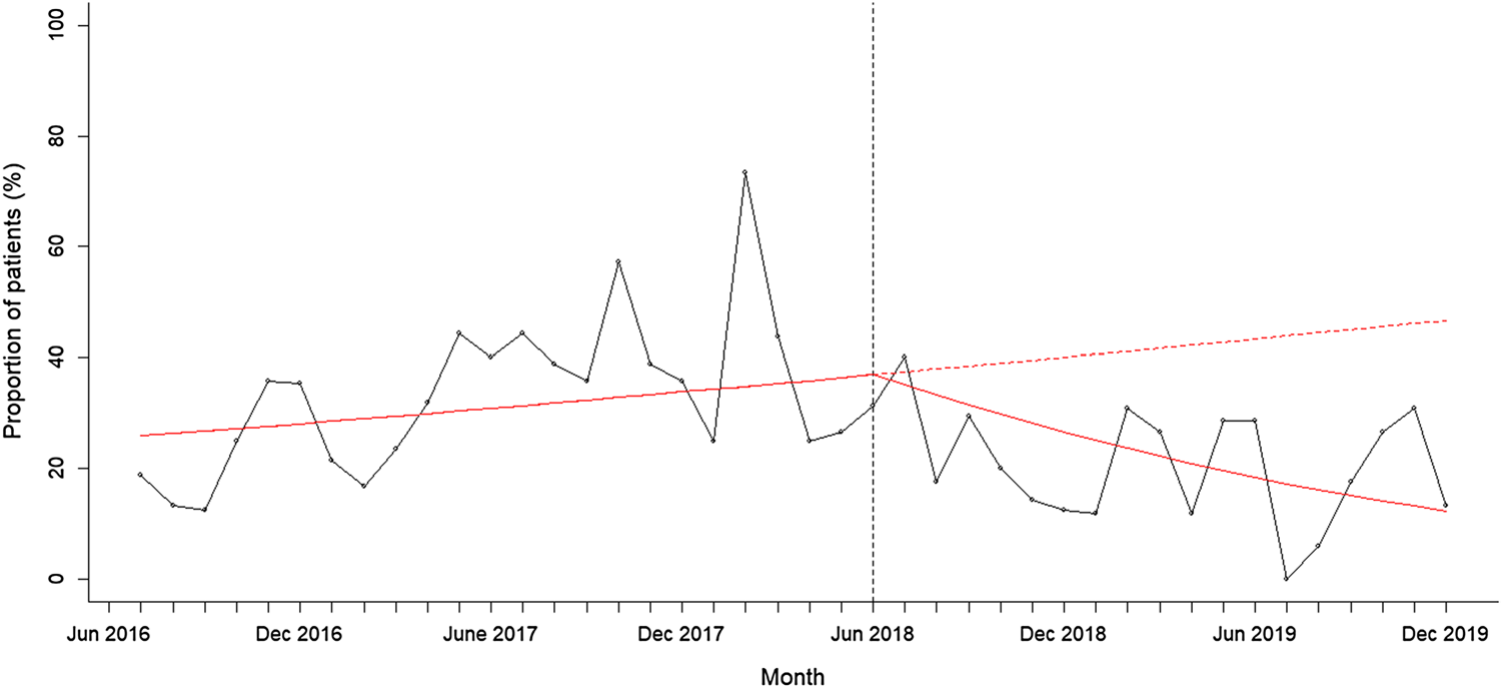
Proportion of patients self-harming at baseline and post intervention. The figure shows the proportion of patients self-harming per month between 1st June 2016 and 31st November 2019 after removing outliers. The intervention was introduced on 1st June 2018 indicated by the vertical line. The solid red line indicates the segmented regression analysis conducted at baseline and at post-intervention. The red dashed line indicates the counterfactual scenario (i.e. projected proportion of patients self-harming if the intervention had not been conducted)

**Fig. 4:**
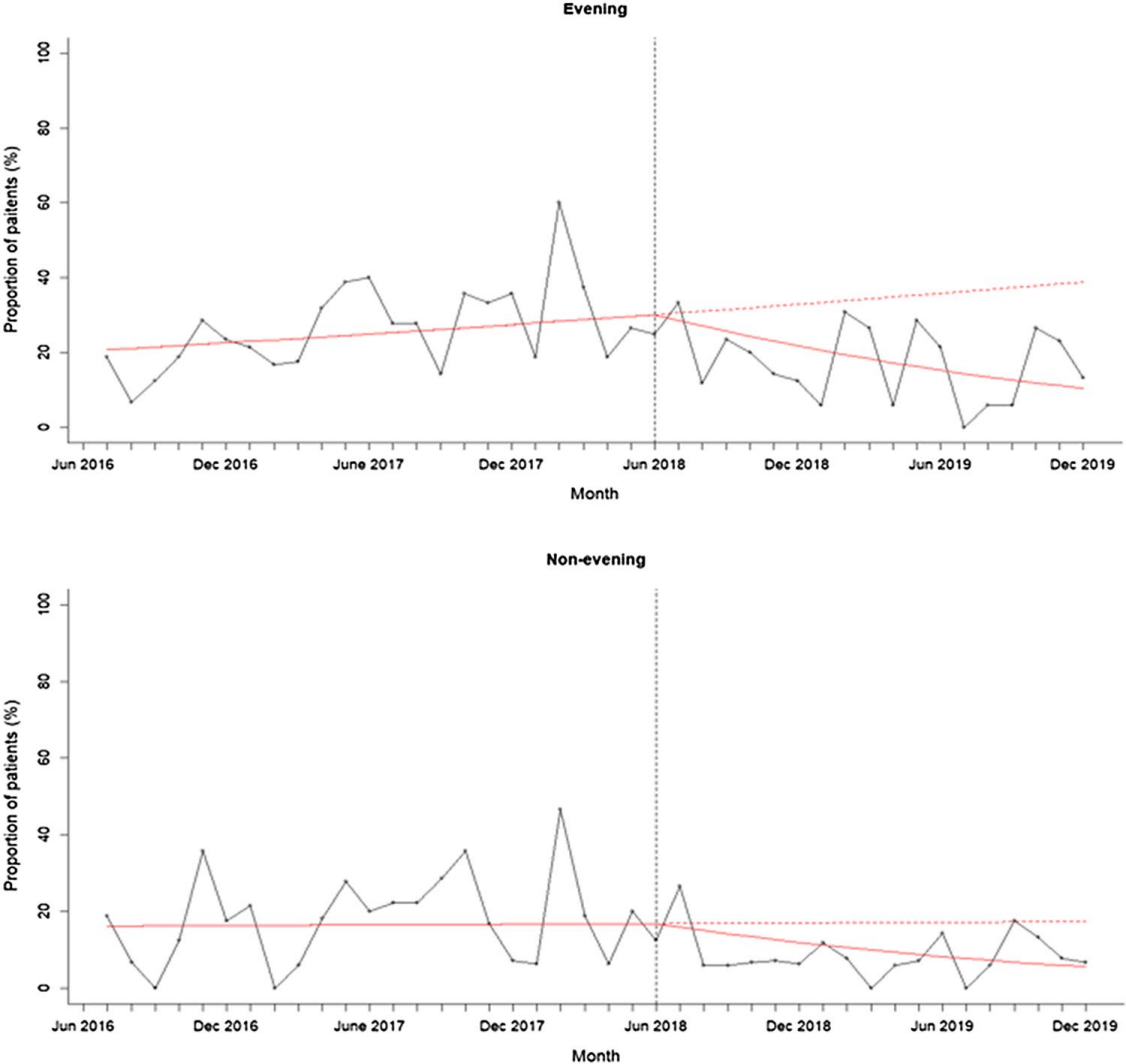
Proportion of patients self-harming at baseline and post intervention split by evening and non-evening period. The figure shows the proportion of patients self-harming per month between 1st June 2016 and 31st November 2019 after removing outliers. Evening refers to 3-11pm and non-evening period refers to any time of day excluding 3-11pm. The intervention was introduced on 1st June 2018 indicated by the vertical time. The solid red line indicates the segmented regression analysis conducted at baseline and at post-intervention. The red dashed line indicates the counterfactual scenario (i.e. projected proportion of patients self-harming if the intervention had not been conducted). The intervention therefore achieved its main effect during the evening periods

A segmented regression analysis without outliers showed that the proportion of patients self-harming was increasing before the intervention and significantly reduced following intervention (see Fig. 3; change in slope – 0.18, 95% CI – 0.16 to – 0.04, *p* = 0.001). When split by time of day, the proportion of patients self-harming per month was also increasing in the evening period before the intervention and significantly reduced after the intervention was introduced (see Fig. 4; change in slope – 0.09, 95% CI – 0.16 to – 0.03, *p* = 0.004). The proportion of patients self-harming per month in the non-evening period was also increasing before the intervention and reduced after the intervention was introduced, but the rate of decline was not significant (change in slope – 0.06, 95% CI – 0.15 to 0.01, *p* = 0.09). This analysis shows that the proportion of patients self-harming significantly reduced after the intervention was introduced, and this effect was driven primarily by a significant reduction in the evening.

When outliers were included in the analysis, the proportion of patients self-harming per month was increasing before the intervention but significantly reduced following intervention (change in slope: – 0.06, 95% CI – 0.13 to – 0.01, *p* = 0.021). The proportion of patients self-harming per month in the evening was also increasing before the intervention and reduced after the intervention was introduced, but this was trending at significance (change in slope: – 0.06, 95% CI – 0.12 to 0.001, *p* = 0.054). The proportion of patients self-harming in the non-evening period also increased before the intervention and reduced after the intervention was introduced, but the rate of decline was not significant (change in slope: – 0.01, 95% CI – 0.09 to 0.06, *p* = 0.704). This indicates that even with the two outlier patients included, the proportion of people self-harming reduced post-intervention and this was driven by a reduction in the evening.

## Discussion

The current study evaluated an intervention that made immediate changes to an adolescent psychiatric inpatient environment to reduce self-harming behaviours with and without suicidal intent. Evenings were identified as a peak time for self-harm incidents occurring on the ward, and similar rates have been reported on adult psychiatric settings [32–34]. An evening-focused intervention was designed with two main components; introducing a regular nursing shift between 3 and 11 pm, and implementing a structured activity programme for weekday evenings. An interrupted time series analysis was conducted to assess the longitudinal effects of the intervention on self-harm in young people. The rate of self-harm was declining at baseline and continued to decline following the intervention, but the rate of decline after the intervention was not significantly different to baseline. Nevertheless, the proportion of adolescents self-harming did significantly reduce following the intervention compared to baseline, even when two patients with numerous self-harm incidents were included in the analysis. The reduction was significantly larger in the evenings compared to the day, indicating that the evening-based interventions were driving the effects. This finding is both important from a therapeutic standpoint for patients and for clinical wards where resources are typically overstretched.

Previous interventions that have made environmental changes to adolescent inpatient settings have either focused on long-term systemic changes with staff training as a main component [51, 52] or have made immediate changes on the ward alongside introducing psychosocial therapies [43, 44]. This includes a recent study which introduced leisure activities for patients on the inpatient ward similar to those introduced in the present study, but in conjunction with an extensive DBT programme [44]. The study found that self-harming behaviour and suicide attempts decreased following the intervention, but a decline in the number of patients self-harming was not reported. It was also not possible to determine the impact of environmental changes on self-harm incidents [44]. The current study goes beyond these studies in showing that immediate environmental changes can reduce the number of adolescent inpatients who self-harm with and without suicidal intent on the ward.

A number of underlying mechanisms and functions of self-harm influenced by the intervention could have led to a reduction in young people self-harming on the ward. Availability of an additional nursing staff at a risky time on the ward could make it easier for nurses to intervene when adolescents begin to show early warning signs of distress [28]. This benefit is likely associated with the availability of nursing staff that have an ongoing relationship with young people on the ward instead of temporary staff that may come on the ward occasionally [28]. In the current intervention, the twilight shifts began to be increasingly filled by regular staff members and this may have led to a reduction in young people self-harming. It is plausible that increased visibility of staff may also reduce anxiety for patients on the ward and, therefore, reduce the likelihood that self-harm is used as a method to seek help. This is supported by the role of interpersonal functions of self-harm [3–5], as well as studies which find that self-harm and other harmful behaviours occur most often in the absence of regular staff [34, 64] and can be reduced by increasing staff visibility on corridors [65]. Clinicians in our study reported that an additional member of staff in the evening helped to alleviate stress, suggesting that presence of more staff helps to improve the general ward atmosphere.

Another component of the intervention was introducing a structured activity programme in the evenings. Since evenings are generally unstructured times of the day on psychiatric wards, some patients may find themselves feeling vulnerable and emotionally distressed during this time and using self-harm as a coping mechanism to regulate negative emotions such as feelings of pain and anger [3, 4, 33, 34, 48, 66]. Meaningful activities in the evening have been suggested as a positive way to distract patients who have negative thoughts and feelings [33, 47, 48, 67], and may help to replace the positive functions associated with self-harm with or without suicidal intent such as sensation-seeking and feelings of gratification [4]. Self-harm is also a private act in young people [36], and evening activities could delay patients from retreating early to their bedrooms, where they are likely to engage in self-harming behaviours alone or behaviours such as brooding which are indicative of suicidal behaviours [32, 48]. Patients admitted on psychiatric wards also report feelings of isolation, restriction and loneliness, and activities offered on the ward may foster positive relationships with other inpatients on the ward and feelings of group cohesion [36, 47], which can likely reduce feelings of isolation for young people and provide short-term relief. Another function of self-harm is the need to form relationship with peers through this behaviour [5, 35], and this is particularly important when adolescents are confined to an inpatient setting. Social activities can help replace this function of self-harm by offering a safe space for inpatients to bond and interact with others on the ward. However, distraction is not always beneficial for adolescents who engage in self-harm [34, 68], perhaps because adolescents may only want to be distracted by activities they enjoy. The fact that less young people self-harmed in the present study could be because patients on the ward were involved in decisions about the evening activity programme before these were introduced on the ward and, therefore, were more likely to engage with these activities. However, the intervention did not have an impact on the most vulnerable patients who self-harmed repeatedly as evident by the outliers. This supports the need for a more cohesive programme of care for patients on psychiatric wards; interventions should attempt to provide both short-term relief from distress by improving the ward environment in conjunction with long-term therapeutic care to reduce self-harm for all adolescent patients.

### The value of an interrupted time series analysis

The current study demonstrates that an interrupted time series method can be used to rigorously evaluate interventions that improve healthcare systems over time when randomisation is not possible [55, 59–61]. Specifically, an interrupted time series analysis can account for any trends that may have existed before the intervention was introduced which is not always possible to detect in a simple pre-post analysis [55, 59, 60]. This is highlighted by our findings, where rates of self-harm did not significantly reduce following the intervention, as the rate of self-harm was already declining on the ward in 2 years preceding the intervention. Better analytical and research techniques have been advocated for interventions that attempt to improve complex healthcare services and systems [44, 69] and an interrupted time series is one approach which should be considered when evaluating health systems interventions over time.

### Limitations and future work

The current findings should be interpreted in light of the limitations of implementing and evaluating this intervention. First, it was not possible to determine which intervention component contributed to a reduction in self-harming behaviour in young people. Despite introducing the structured activity programme after the twilight shifts had been embedded into practice, the time between these interventions was not sufficient to be analysed separately using an interrupted time series analysis. Second, while all patients were diagnosed by one psychiatrist using a standard clinical assessment, no validated diagnostic interview was used. This could make it difficult to compare diagnoses of patients in the current study with patients from other interventions. In addition, two patients self-harmed on the ward several times repeatedly during the study period and were considered outliers for the purpose of analysis. However, it was not clear whether the high rate of self-harm in these patients was due to individual factors or other aspects of the ward. Further work is still needed to reduce self-harm in high-risk adolescents on psychiatric wards. It was also not possible to determine whether the intervention reduced the number of people self-harming with or without suicidal intent, as intention was not reported in routinely collected incident data. It is likely, however, that the intervention had an impact on both types of self-harming behaviour, given that non-suicidal self-harm and self-harm with suicidal intent often co-occur and are closely related [7, 26, 70]. We recommend that in the future UK healthcare organisations should be encouraged to state the intent of self-harm when reporting these patient incidents, as this will be informative both for clinical teams and when reporting these incidents more widely. Another limitation is that the mechanisms of the intervention could only be inferred based on a limited understanding of the contributory factors of a psychiatric ward environment on adolescent self-harming behaviour. More research is needed so that interventions can be developed and targeted more effectively. It was also not possible to determine whether self-harm was influenced by how the intervention was implemented on the ward, such as what the staff did during the evening shifts, the type of activities that were conducted and even which inpatients took part in these activities. The aim of the intervention, however, was to identify the main intervention components which could be implemented and adapted based on the local context. Conducting an interrupted time series analysis further helped to minimise any impact on self-harm due to daily fluctuations on the ward and helped demonstrate the broader impact of the intervention over time.

## Conclusion

Increased staff availability and introducing a structured activity programme during evenings on an adolescent psychiatric ward helped to reduce the proportion of young people who self-harm. This is an important finding both from a therapeutic standpoint and for overstretched healthcare services, where support can be provided to the most vulnerable patients. The study shows that in mental health, as in other safety–critical settings, changes to the environment and the organisation of care should be considered alongside direct therapeutic interventions when seeking to improve patient safety. An interrupted time series analysis should also be considered when evaluating interventions to health systems over time.

## Electronic supplementary material

Below is the link to the electronic supplementary material.

Supplementary material 1 (DOCX 12 kb)

## Data Availability

Due to sensitive patient information, data will not be made publicly available.
