# Supplementary material for: Environmental changes to reduce self-harm on an adolescent inpatient psychiatric ward: an interrupted time series analysis
Source: Eur Child Adolesc Psychiatry. 2020 Jul 27;30(8):1173–86. doi: 10.1007/s00787-020-01607-3 (PMC8310847; doi:10.1007/s00787-020-01607-3)
Supplement: Supplementary file 1 — Supplementary material 1 (DOCX 12 kb) [file 787_2020_1607_MOESM1_ESM.docx]

Appendix

**Table A.1** Use of temporary agency staff as a percentage of total staff use, pre and post-intervention

|  | **Agency staff usage** | |
| --- | --- | --- |
|  | **Mean (SD)** | **Range** |
| **Pre-intervention** | 20.29 (4.57) | 12.4 – 29.3 |
| **Post-intervention** | 5.04 (2.91) | 0.3 – 10.7 |

Pre-intervention dates: 1^st^ June 2016 to 31^st^ May 2018; Post intervention dates: 1^st^ June 2018 to 31^st^ November 2019
